# Identifying the Specific Root Microbiome of the Hyperaccumulator *Noccaea brachypetala* Growing in Non-metalliferous Soils

**DOI:** 10.3389/fmicb.2021.639997

**Published:** 2021-05-14

**Authors:** Soledad Martos, Sílvia Busoms, Laura Pérez-Martín, Mercè Llugany, Catalina Cabot, Charlotte Poschenrieder

**Affiliations:** ^1^Plant Physiology Laboratory, Bioscience Faculty, Universitat Autònoma de Barcelona, Bellaterra, Spain; ^2^Department of Biology, Universitat de les Illes Balears, Palma, Spain

**Keywords:** high-throughput sequencing (deep sequencing), *Noccaea brachypetala*, root-associated microbial communities, Zn/Cd hyperaccumulator, non-metalliferous soil

## Abstract

*Noccaea brachypetala* is a close relative of *Noccaea caerulescens*, a model plant species used in metal hyperaccumulation studies. In a previous survey in the Catalan Pyrenees, we found two occidental and two oriental *N. brachypetala* populations growing on non-metalliferous soils, with accumulated high concentrations of Cd and Zn. Our hypothesis was that the microbiome companion of the plant roots may influence the ability of these plants to absorb metals. We performed high-throughput sequencing of the bacterial and fungal communities in the rhizosphere soil and rhizoplane fractions. The rhizobiomes and shoot ionomes of *N. brachypetala* plants were analyzed along with those from other non-hyperaccumulator Brassicaceae species found at the same sampling locations. The analyses revealed that microbiome richness and relative abundance tended to increase in *N. brachypetala* plants compared to non-hyperaccumulator species, regardless of plant location. We confirmed that the root compartment is a key factor in describing the community composition linked to the cohabiting Brassicaceae species, and the rhizoplane fraction contained the specific and rare taxa associated with each species. *N. brachypetala* plants harbored a similar relative abundance of fungi compared to the other plant hosts, but there was a notable reduction in some specific taxa. Additionally, we observed an enrichment in the hyperaccumulator rhizoplane of previously described metal-tolerant bacteria and bacteria involved in nitrogen cycling. The bacteria involved in the nitrogen cycle could contribute indirectly to the hyperaccumulator phenotype by improving soil quality and fertility. Our results indicate that *N. brachypetala* captures a particular prokaryotic community from the soil. This particular prokaryotic community may benefit the extraction of metal ions and/or improve plant nutrition. Our research identified satellite groups associated with the root niche of a hyperaccumulator plant that may assist in improving biological strategies in heavy metal remediation.

## Graphical Abstract

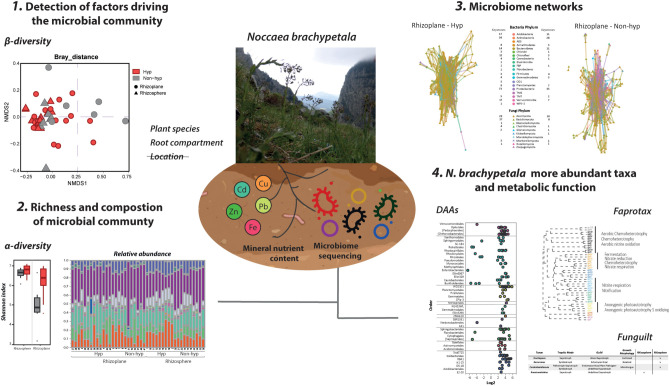

## Introduction

Metal-rich soils are harsh environments for establishing life. However, metallophytes have evolved different strategies to survive under these restrictive growth conditions. Among these plant species, hyperaccumulators harbor metal concentrations within aerial tissues at levels far exceeding those present in their native soil. Without showing significant signs of toxicity, they can achieve 100-fold higher shoot metal concentrations than non-hyperaccumulator plants (Van der Ent et al., 2013). Hyperaccumulation appears to have evolved several times within vascular plants, and the Brassicaceae family contains a high percentage of the total hyperaccumulating taxa (Kramer, 2010). *Noccaea caerulescens* (formerly *Thlaspi caerulescens*) belongs to this family and is one of the best-known hyperaccumulators of Cd, Ni, and Zn (Assunçao et al., 2003). This genus contains several species, including *Noccaea praecox* (Likar et al., 2010), *Noccaea camlikensis* (Aksoy et al., 2015), *Noccaea brachypetala* (Martos et al., 2016), *Noccaea kovatsii* (Misljenovic et al., 2020), and *Noccaea japonica* (Nishida et al., 2020), which have also been described as metal hyperaccumulators. Interestingly, metallophytes are not restricted to metalliferous soils, and it has been suggested that Zn/Cd/Pb hyperaccumulation can evolve on non-metalliferous lands (Besnard et al., 2009). When growing on common soils, the metal concentrations in their shoots can achieve unusually high levels. The most popular theory suggests the evolutionary advantage of this behavior is an increased resistance to biotic stresses (Poschenrieder et al., 2006; Cappa and Pilon-Smits, 2014).

The ability to mobilize, chelate, and transport metals is directly linked to soil metal bioavailability and the genetic background of the plant. However, recent research highlights that plant-associated microorganisms can significantly enhance metal availability. The soil microbial community may greatly contribute to the hyperaccumulator phenotype (Visioli et al., 2015a). Various studies have reported a high diversity of microbes in the rhizosphere and endosphere (Thijs et al., 2017). Under unfavorable conditions of metalliferous soils, rhizosphere microorganisms may increase or decrease metal availability (Farinati et al., 2011). Previous studies have demonstrated both effects in *Noccaea* with root-bacteria favoring metal uptake (Whiting et al., 2001), whereas fungal mycorrhiza reduced plant absorption (Vogel-Mikus et al., 2006). However, a proper cohort of microbes may increase the potential of hyperaccumulators to extract metals. Various studies have confirmed that specific bacteria promote the uptake of Ni in *N. caerulescens* (Aboudrar et al., 2013; Visioli et al., 2014, 2015b). Therefore, rhizosphere-associated microbes from hyperaccumulators are a potential source of genotypes for biotechnological applications in phytoremediation and agriculture (Rajkumar et al., 2012). Characterization of the rhizobiome of hyperaccumulators is needed to enable the development of these applications. Despite this, a limited number of hyperaccumulator species have been investigated (Alford et al., 2010; Ma et al., 2011; Sessitsch et al., 2013). Within the genus *Noccaea*, the rhizosphere bacteria of *Noccaea goesingense* were characterized by Idris et al. (2004), and mycorrhizal colonization in *N. praecox* has been investigated (Pongrac et al., 2007). The microbiomes of other *Noccaea* hyperaccumulator species remain unknown.

Recent metagenomic studies revealed that soil properties are the major drivers for the differentiations in the assembly of root communities in *Arabidopsis thaliana* and in some related species, including the Cd-Zn hyperaccumulator, *Arabidopsis halleri* (Bulgarelli et al., 2012; Lundberg et al., 2012; Schlaeppi et al., 2014; Glynou et al., 2018). Plant genotype research remains conflicted. Some authors affirm that plant genotype is a key factor in microbiome assembly (Burns et al., 2015; Li et al., 2018); others do not consider this essential (Glynou et al., 2018; Macia-Vicente et al., 2020). Proximity to the root is also considered a relevant parameter that increases soil microbiota diversity and structure (Martinez-Diz et al., 2019; Macia-Vicente et al., 2020; Thiergart et al., 2020). Several studies have shown that plant exudates can favor specific microbiome communities mutualistically (Bulgarelli et al., 2013; Stringlis et al., 2018). Exudated organic compounds nurture rhizomicrobiota, allowing the root communities to modulate the plant phenotype. A well-reported case revealed an increased metal tolerance in plants associated with a metal-tolerant microbiome (Luo et al., 2014; Visioli et al., 2015a). However, the potential role of microbiota from non-metalliferous soils to the hyperaccumulation phenotype remains unknown. In the last few years, vast progress has been made in elucidating the composition and dynamics of root communities in the Brassicaceae family. However, additional studies in natural conditions are required. We investigated the impact of plant identity, location, and root compartment on the microbial diversity and community structure of the hyperaccumulator *N. brachypetala* and other Brassicaceae species (non-hyperaccumulators) coinhabiting undisturbed and non-metalliferous soils of the Catalan Pyrenees.

## Materials and Methods

### Plant and Soil Sampling

**FIGURE 1:**
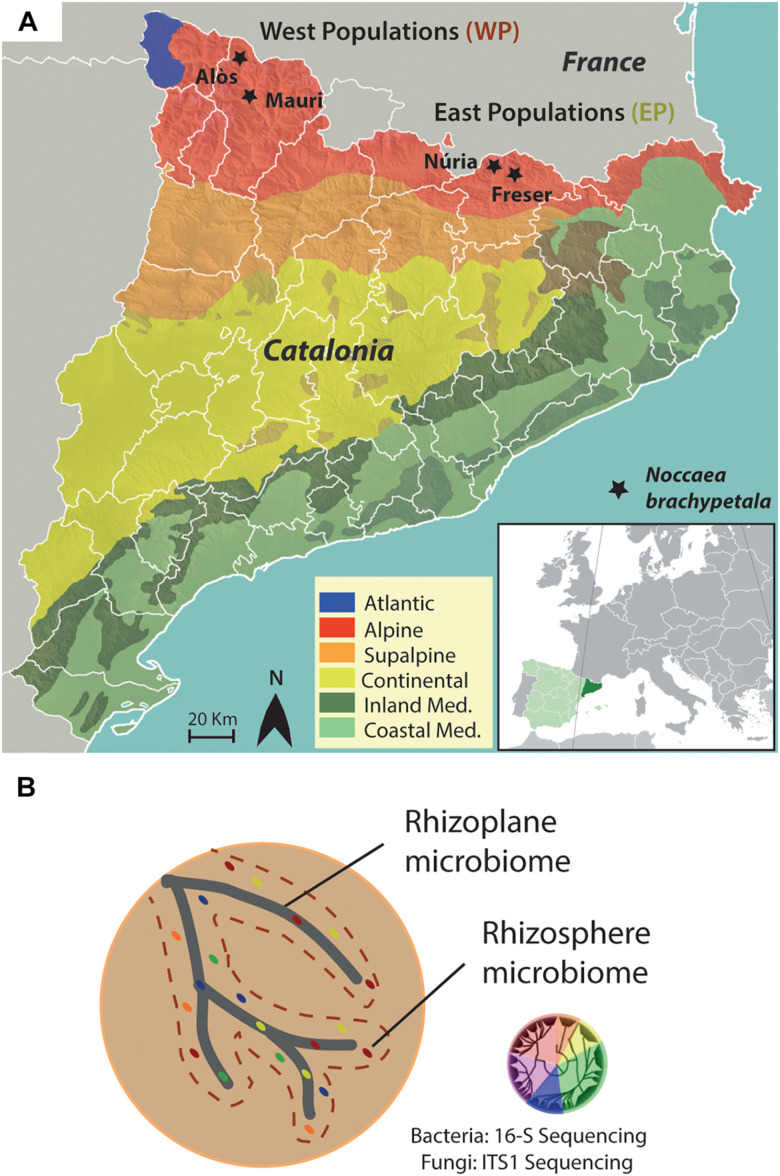
Experimental design. **(A)** Climatic map of Catalonia with the sampling locations of plants and soils indicated by stars. Western populations are composed form locations of Alòs and Mauri, whereas Eastern populations are from Núria and Freser. **(B)** Schematic drawing of the analyzed compartments of the root. DNA of the microbiome from the rhizoplane and rhizosphere was extracted followed by 16S and ITS sequencing of the bacteria and fungi, respectively.

Plant and soil materials were collected from four sampling sites in the Catalan Pyrenees. Two sites were located in the Pallars region (Mauri and Alòs), and the other two were in the Ripollès region (Freser and Núria). All sites were located in the alpine biome of the Catalan Pyrenees (Figure 1A). Plants from Mauri and Aneu were grouped as Western populations (WPs), and Freser and Nuria plants were grouped as the Eastern populations (EPs). Both locations are separated by more than 150 km. The geographic coordinates of each sampling site are listed in Table 1.

**TABLE 1:** Sampled plants for the rhizosphere microbioma sequencing.

	Brassicaceae species	DNA extracted compartment	Location
1	*Noccaea brachypetala*	R, RP1, RP2	Alòs (Western population) 42° 45′ 36.792″ N, 1° 4′ 13.8″ E
2	*N. brachypetala*	R, RP1, RP2	
3	*N. brachypetala*	R, RP1, RP2	
4	*N. brachypetala*	R, RP1, RP2	
5	*N. brachypetala*	R, RP1	
6	*Capsella bursa-pastoris*	R, RP1	
7	*Arabis* sp.	R, RP1	
8	*Cardamine impatiens*	R, RP1	
9	*N. brachypetala*	R, RP1, RP2	Mauri (Western population) 42° 34′ 44.508″ N, 1° 0′ 30.528″ E
10	*N. brachypetala*	R, RP1, RP2	
11	*N. brachypetala*	R, RP1, RP2	
12	*N. brachypetala*	R, RP1	
13	*Arabis* sp.	R, RP1	
14	*N. brachypetala*	R, RP1, RP2	Núria (Eastern population) 42° 21′ 52.164″ N, 2° 10′ 5.88″ E
15	*N. brachypetala*	R, RP1, RP2	
16	*Capsella* sp.	R, RP1	
17	*Iberis* sp.	R, RP1	
18	*N. brachypetala*	R, RP1, RP2	Freser (Eastern population) 42° 22′ 54.624″ N, 2° 12′ 56.268″ E
19	*Iberis* sp.	R, RP1	
20	*Arabis* sp.	R, RP1	

*Genomic DNA has been extracted from the rhizosphere soil (R) and rhizoplane (RP1 and RP2).*

At each site, *N. brachypetala* specimens [previously confirmed as Cd/Zn hyperaccumulators (Martos et al., 2016)] and other non-hyperaccumulator Brassicaceae species growing beside the specimens were also collected. All collectors wore gloves. A list of sampled species is provided in Table 1. Selected individuals were excavated with their rhizosphere soil without disturbing the plant root system. Plants were immediately transferred into polyethylene bags to avoid desiccation. From each site, bulk soil (five replicates per site) exempt from root or plant debris was collected at a depth of 10 cm using a 7-cm diameter Edelman drill (Eijkelkamp, Giesbeek, Netherlands). All collected plant and soil samples were refrigerated in a portable icebox and immediately transported to the laboratory for further processing.

### Analysis of Metal Content From Soil and Plant Samples

Field-collected plants were separated into aerial and below ground tissue. All aerial tissue was carefully washed with distilled water and oven-dried at 60°C for 2 days. Dried shoots (three replicates/plant species) were finely powdered using a mortar and pestle. In parallel, collected bulk soils (five replicates per sampling site) were homogenized through a 2-mm sieve. Approximately 1 g of sieved bulk soil and 0.1 g of powdered shoots were acid-digested (HNO_3_: H_2_O_2_ 69%: 30%, 5:2 vol/vol) in a hot-block digestion system (SC154-54-Well Hot Block^TM^, Environmental Express, SC, United States). The concentrations of the selected metal elements (Cd, Cu, Pb, and Zn) and additional elements (Ca, Co, Fe, K, Mg, Mn, Mo, Na, P, and S) were determined by inductively coupled plasma (ICP) mass spectrometry (Perkin Elmer Inc., ELAN 6000, MA, United States) or ICP–optical emission spectrometry (Thermo Jarrell-Ash, model 61E Polyscan, England) (Bech et al., 2012). Internal references of the certified material BCR 62 *Olea europaea* and CRM 142 R Light Sandy Soil were included for quality control.

Statistical differences among groups in the element content of the soils or leaves were assessed using analysis of variance (two-way ANOVA). Normal distribution, homoscedasticity, and the independence of errors were checked prior to analyses. Data were log-transformed when necessary. Multiple comparisons of group means were analyzed by Tukey honestly significant difference using JMP^®^ (version 13.0, SAS Institute Inc., 1989-2019).

### Sampling of Rhizosphere and Rhizoplane Compartments

The soil adhering to the roots of each sampled specimen was separated into two fractions: rhizosphere and rhizoplane (Figure 1B). The sampling method for the rhizosphere and rhizoplane compartments followed the protocols described by Barillot et al. (2013) and Edwards et al. (2015), with minor modifications. In brief, the rhizosphere fraction was manually removed by vigorously shaking for 2 min until approximately 1 mm of soil was firmly attached to the roots. The rhizosphere fraction was suspended in 50 mL of 0.9% NaCl solution. Roots were then placed into sterile 100-mL tubes containing 50 mL of 0.9% NaCl solution and washed by manually shaking for 2 min (RP1 fraction). Roots were subsequently immersed in 0.9% NaCl solution and 0.01% Tween 80 and then washed by manually shaking for 2 min (RP2 fraction). Rhizosphere and rhizoplane RP1 and RP2 suspensions were then homogenized with gentle agitation (300*g*, OVAN Orbital Midi OM10E shaker, 25°C, 90 min) followed by centrifugation (5,000*g*, 25°C, 10 min) in sterile 100-mL tubes to concentrate soil particles into a pellet. Then, the supernatant was filtered through a 1-mm sieve to remove suspended residuals. Fractions were frozen in liquid nitrogen and stored at −80°C until required for DNA extraction.

### Library Generation and Sequencing of Bacterial and Fungal DNA Regions

The total genomic DNA from the rhizosphere and rhizoplane compartments (approximately 200 mg) of the *N. brachypetala* specimens and the non-hyperaccumulator Brassicaceae species was extracted using the NucleoSpin^®^ Soil kit (Macherey-Nagel GmbH & Co. KG, Düren, Germany), according to the manufacturer’s instructions. Extracted DNA was purified using the QIAamp DNA Stool Mini Kit (Qiagen, West Sussex, United Kingdom) and then quantified using the Picofluor^TM^ method (PicoGreen dsDNA Quantitation Reagent, Molecular Probes, Inc., Eugene, OR, United States). Quality was measured using Nanodrop 2000 (Thermo Fisher Scientific, DE, United States) and confirmed by the OD A_260_/A_280_ between 1.8 and 2.0.

The prokaryotic 16S ribosomal RNA gene (16S rRNA) and the internal transcribed spacer (ITS) were the barcodes used for the metagenomic study of the bacteria and fungi, respectively. Microbial populations were obtained by high-throughput sequencing, targeting the V3–V4 region of 16S rRNA and ITS1 of the ITS region, using the MiSeq^®^ Reagent Kit v2 (500 cycle) (MiSeq, Illumina, San Diego, CA, United States). The V3–V4 region was amplified using primers 341F (5′-CCTACGGGAGGCAGCAG-3′) and 806R (5′-GGACTACHVGGGTWTCTAAT-3′). The ITS1 region was amplified using primers ITSF (5′-CTTGGTCATTTAGAGGA AGTAA-3′) and ITS2 (5′-GCTGCGTTCTTCATCGATGC-3′).

### Quality Control of Sequencing Data

Sequence reads of both datasets (16S and ITS) from the MiSeq Illumina^®^ system were processed using QIIME v.1.9.1 (Caporaso et al., 2010). The quality filter of the already demultiplexed sequences was performed at a maximum unacceptable Phred quality score of Q20. The resulting reads were clustered into operational taxonomic units (OTUs) using uclust with 97% sequence similarity and the subsampling pick open reference method (Rideout et al., 2014) at 10% of the subsampled sequences. Seven samples with number of reads fewer than 5,000 or a percentage of chimeras higher than 70% were removed from the analysis (Supplementary Dataset 1). Rarefaction curves showed that 90.7% of the sequenced samples achieved a plateau zone, indicating that no additional sequencing effort was required (Supplementary Figure 1). After filtering, a total of 4,752,917 high-quality sequences were obtained from 43 samples: 2,442,668 from the 16S region (bacteria) and 2,310,249 sequences from the ITS region (fungi) (Supplementary Dataset 1). The media range of sequence reads per sample was 56,806 for bacteria and 53,726 for fungi.

Representative sequences were assigned to taxonomic groups using the bacterial 16S GreenGenes v.13.8 reference database at a 90% confidence threshold and sequence alignment using QIIME. The taxonomy of fungi/oomycetes was assigned to full ITS sequences from the UNITE_v2020 database (Nilsson et al., 2018) using the DADA2 software package (Callahan et al., 2016). To reduce artifacts in the dataset, we removed singletons, OTUs with relative abundances across all samples less than 0.005% (Bokulich et al., 2012), and OTUs assigned as chloroplasts or mitochondria. The OTU table was resampled to the minimum number of sequences per sample (Weiss et al., 2017), leading to a total of 3,110 OTUs in the bacterial domain and 1,060 OTUs in the fungal domain.

### Diversity Metrics and Relative Abundance

Output data from QIIME and DADA2 were merged and used to obtain an OUT Phyloseq object generated with R (McMurdie and Holmes, 2012). The α diversity was calculated using the Shannon index. The Kruskal–Wallis significance test was used for all pairwise combinations (between root compartments and plant species).

Based on the OTU relative abundance, a β-diversity assessment was performed using Bray–Curtis metrics. The resulting distance/dissimilarity matrices were subjected to ordination using principal coordinate analysis.

Non-metric multidimensional scaling was analyzed based on the Bray–Curtis distances between samples using the Vegan package (Oksanen et al., 2013). The significance of each factor [root compartment, location, plant species, and extraction methodology (RP1, RP2)] and all the edaphological variables (nutrient mineral content) in the microbial composition were assessed by permutational multivariate ANOVA (PERMANOVA) using the Adonis function (R package vegan). Prior to analysis, edaphological data were standardized (*Z* scores) to avoid bias caused by the order of magnitude. All factors were independently analyzed. Factors that did not condition the microbiome of any analyzed group were not further analyzed.

The assessment of the taxonomic composition was performed at the phylum level using staked plots. To detect differences in bacterial and fungal community composition, we performed ANOVA pairwise comparisons between root compartments and plant species.

### Co-occurrence Network

The OTU phyloseq object was converted into an adjacency matrix based on covariance by the spiec.easi() function of the *SpiecEasi* package (version 1.0.7) and using MB’s neighborhood selection and minimum λ threshold set to 0.01, for all networks. Bacteria and fungi domains were analyzed together, and independent networks were constructed for each root compartment and plant species. Fundamental OTU networks (keystones) were the highest “betweenness” and “node count” in more than 50% of the distribution. Each adjacency matrix was then converted into an *igraph* object and visualized as a network using the adj2igraph() and plot network() functions in SpiecEasi.

### Differential Abundance Analysis

To establish which taxa were associated more commonly with *N. brachypetala* compared to non-hyperaccumulator species in each compartment, a differential abundance analysis (DAA) was performed using the DESeq2 package. This method models the observed abundances using negative binomial distribution after normalizing data with the corresponding scaling methods to account for differences in sampling fractions, recommended for small datasets (< 20 samples per group) (Paulson et al., 2013; Weiss et al., 2017; Lin and Peddada, 2020). Plot visualization was performed using the ggplot package.

### Metabolic and Functional Analysis

To determine the metabolic potential of the *N. brachypetala* bacterial communities that were differentially abundant, a predictive functional analysis of bacterial communities was performed using FAPROTAX software (Louca et al., 2016). Similarly, functional annotation of the *N. brachypetala–*specific fungal communities was obtained using FUNGuild (Nguyen et al., 2016).

## Results

### Soil and Leaf Mineral Content

**FIGURE 2:**
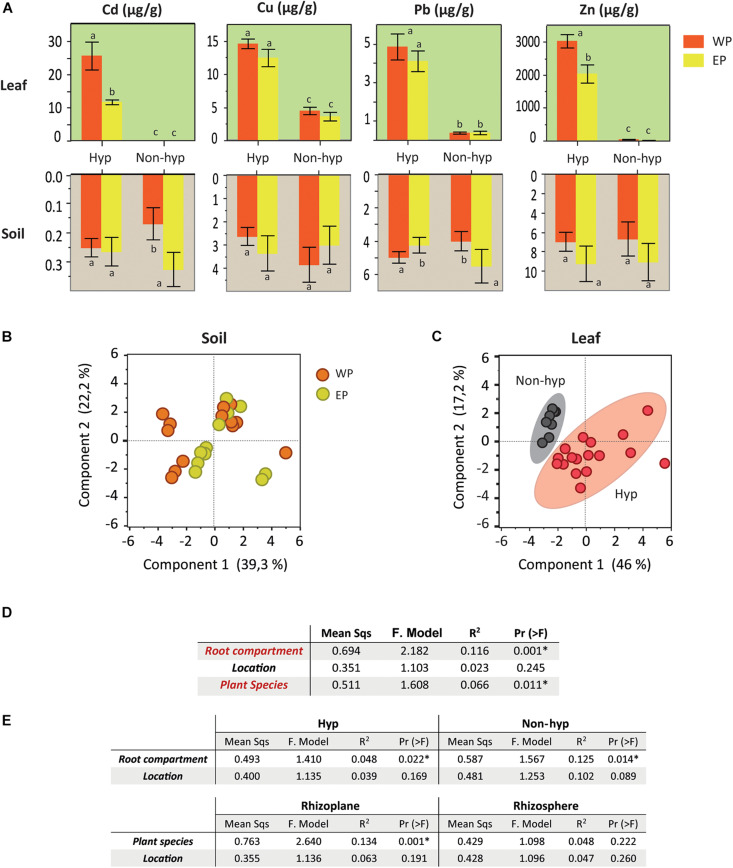
Soil and leaf nutrient mineral content and factors potentially influencing the microbiome. **(A)** Bar plots for soil and leaf metal content of hyperaccumulator and non-hyperaccumulator plants from Western (WP in orange) and Eastern populations (EP in yellow). Error bars correspond to the standard error. Different letters indicate statistically significant differences among treatments at *P* < 0.05. **(B)** Principal component analysis of the soil mineral content in the Western (orange dots) and Eastern populations (yellow dots). **(C)** Principal component analysis of the leaf mineral content. Hyperaccumulator plants are clustered in red and non-hyperaccumulator species in gray. **(D)** Summarized table of the PERMANOVA on the effect of the soil mineral content, root compartment, plant species, and location on the microbiome community (grouping bacteria and fungi). **(E)** Summarized table of the PERMANOVA on the effects of location and root compartment or plant species on the microbiome community (grouping bacteria and fungi).

The analyses of the metal concentrations catalogued all the soil samples as non-metalliferous. The bulk soil collected with the different plant species revealed slight differences in the metal contents according to their location (EP/WP) (Figure 2A and Supplementary Dataset 2). For example, WPs of *N. brachypetala* accumulated higher levels of Cd and Zn compared to the EPs. However, the principal component analysis (PCA) of the soil samples did not differentiate the samples into location clusters (Figure 2B). The PCA of the ionomic data identified the hyperaccumulation trait as the key clustering factor in the samples of *N. brachypetala* with the other Brassicaceae species in independent groups (Figure 2C). The metal content in the leaves of *N. brachypetala* was 200 times higher for Cd and more than 100 times higher for Zn than for the non-hyperaccumulator species (Figure 2A and Supplementary Dataset 2). The data confirmed the Cd and Zn hyperaccumulator ability of *N. brachypetala*. Other elements, such as K, Cu, P, and Pb, were also increased in *N. brachypetala* leaves but at lower levels (from 1.5 to 10 times higher levels, Supplementary Dataset 2).

### Root Compartment and Plant Species Condition the Microbial Composition

The statistical analysis (PERMANOVA) of the OTUs from the bacteria and fungi domains revealed that the root compartment and plant species were relevant factors in explaining the variability of microbiome data (Figure 2D). However, the weight of the factor “plant location” was not significant. The effect of location was irrelevant even when plant species and root compartments were analyzed separately (Figure 2E).

Additionally, we evaluated the effect of the extraction method on the rhizoplane. The multivariate ANOVA confirmed no statistically significant difference between the extraction methods (RP1 and RP2) (Supplementary Datasets 1,2). Therefore, we selected root compartment and plant species as factors to investigate their effects on microbial diversity, structure, and composition.

### High Influence of Root Compartment and Plant Species in the Bacterial Community

**FIGURE 3:**
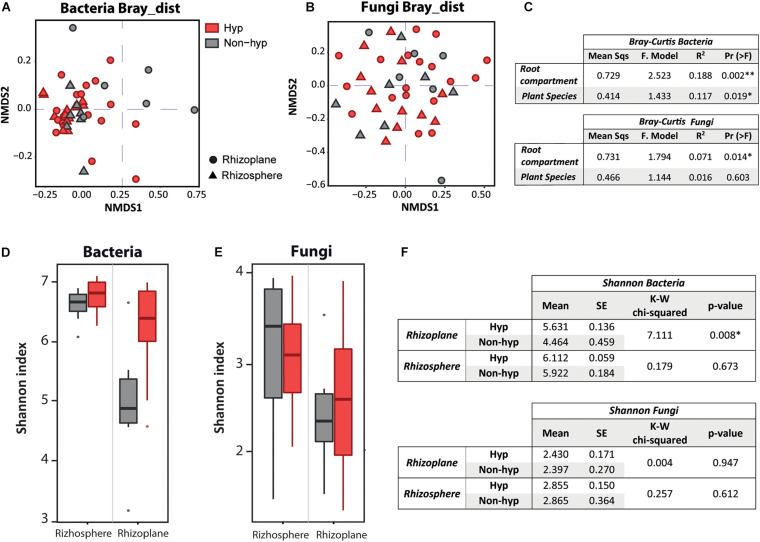
Factors influencing the bacterial and fungal community and diversity. Non-metric multidimensional scaling visualization of the Bray–Curtis results of the bacterial domain **(A)** and fungal domain **(B)**. Plant species are differentiated by color (red is the hyperaccumulator; gray is the non-hyperaccumulator), and root compartments by shape (dots represent the rhizoplane; triangles represent the rhizosphere). **(C)** Summarized table of the PERMANOVA of the Bray–Curtis distances on the effect of root compartment and plant species for the bacterial and fungal domain, separately. **(D)** α Diversity boxplot of the bacterial domain. **(E)** α Diversity boxplot of the fungal domain. The Shannon index divided by root compartment and plant genotype. The *x* axis indicates root compartment, whereas plant genotype is differentiated by color (red is *N. brachypetala*; gray represents non-hyperaccumulator species). **(F)** Summarized table of the Kruskal–Wallis analysis from the Shannon index on the effect of root compartment of the bacterial and also the fungal domain.

Root-associated microbes were divided into bacteria and fungi to investigate factors that could drive the microbial community within each domain (Figures 3A–C). The root compartment and plant species in the bacterial composition possessed larger proportions of variation (19%, *P* < 0.01, and 12%, *P* < 0.05, respectively) (Figures 3A,C). In contrast, the Bray–Curtis analysis indicated that the root compartment was a unique and significant factor describing the fungal composition, whereas the plant species was not relevant (Figures 3B,D).

The α-diversity boxplots (using the Shannon index) showed higher diversity in the rhizosphere than in the rhizoplane compartment for both microbial groups (Figures 3D,E). Moreover, rhizoplane diversity in the bacteria community was increased in *N. brachypetala* (hyperaccumulator) compared to the non-hyperaccumulator species (*P* < 0.01, Figure 3F). Fungal diversity did not vary between plant species (*P* > 0.05, Figure 3F).

### More Complex Network in the Rhizoplane of *N. brachypetala*

**FIGURE 4:**
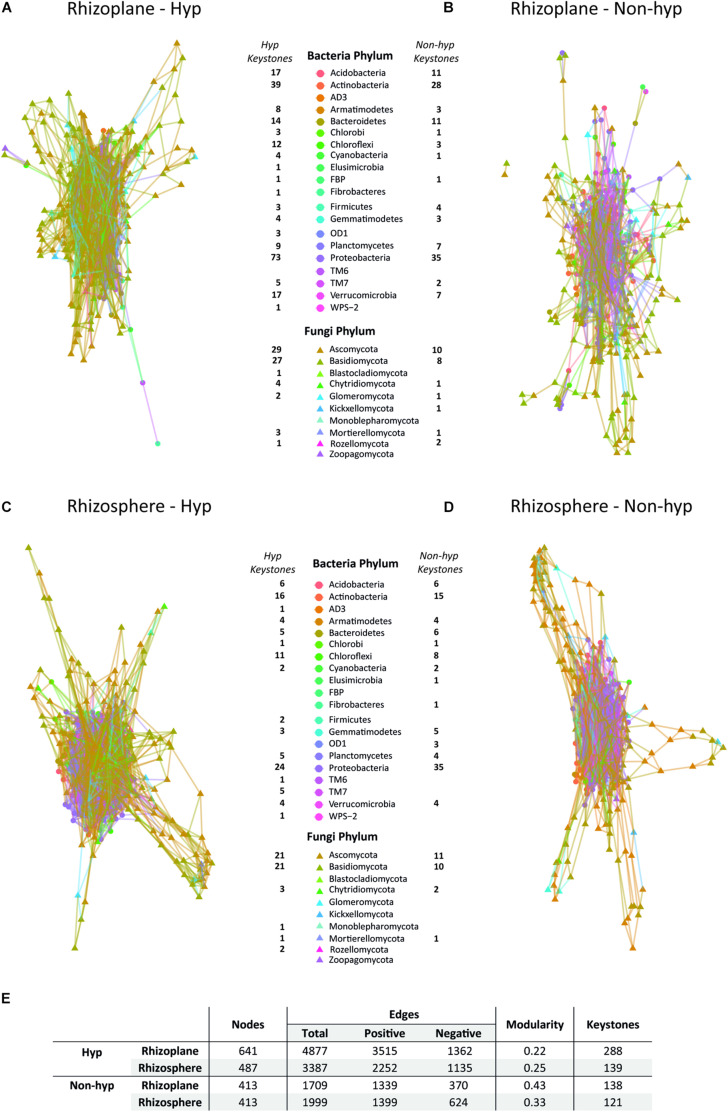
Co-occurrence network. Potential relationship among the bacterial and fungal OTUs in the rhizoplane **(A,B)** and the rhizosphere **(C,D)** of *N. brachypetala* (Hyp) **(A,C)**, and the non-hyperaccumulator species (Non-hyp) **(B,D)**. **(E)** Table with the main parameters of the co-occurrence networks.

The interaction patterns of the overall microbiome were analyzed and revealed that the hyperaccumulator networks were globally more interconnected than the non-hyperaccumulator networks (Figures 4A–D). *N. brachypetala* established more connections among phyla, as indicated by the increased number of total edges when compared to the other species (Figure 4E). Interestingly, the rhizoplane of the hyperaccumulator displayed the most complex interaction network with more nodes and edges (Figures 4A,E). In all the represented networks, the central module was composed of bacterial phyla (circles) surrounded by fungal phyla (triangles) (Figures 4A–D).

The most important OTUs for the overall structure (keystones) were identified. The *N. brachypetala* rhizoplane presented the highest number of keystones. Proteobacteria and Actinobacteria species and Ascomycota and Basidiomycota species may play an important role in plant-microbial feedback as they possess the largest number of keystones in the four networks analyzed (Figures 4A–D).

### Higher Relative Abundance of Bacteria and Fungi in *N. brachypetala* With Some Exclusive Taxa

**FIGURE 5:**
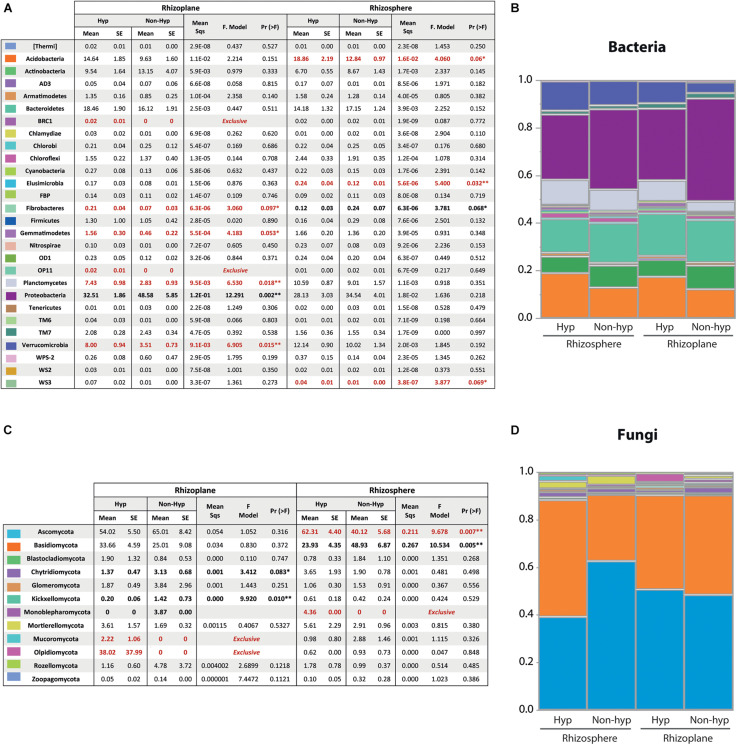
Bacterial and fungal community composition and relative abundance. **(A)** Means and standard errors (SE) of the relative abundances of the bacteria domain at phylum level. Differences and summarized results of an ANOVA pairwise comparison between *N. brachypetala* and non-hyperaccumulators at each soil compartment were calculated by the log2 ratio. **(B)** Bacterial relative abundance at the phylum level in a staked plot. The *x* axis indicates root compartment and plant genotype. **(C)** Means and standard errors (SE) of the relative abundances at phylum level on the fungi domain. Differences and summarized results of an ANOVA pairwise comparison between *N. brachypetala* and the non-hyperaccumulators at each soil compartment calculated by the log2 ratio. **(D)** Fungal relative abundance (at the phylum level) in a staked plot. The *x* axis indicates the root compartment and plant genotype.

To detect differences in the composition of bacterial and fungal communities, the absolute abundance was transformed into relative values. Data were clustered by phyla and grouped by hyperaccumulator/non-hyperaccumulator and rhizosphere/rhizoplane (Figure 5). The relative abundance of each sample is shown in Supplementary Figure 3. The bacterial domain was dominated by six major phyla: Acidobacteria, Actinobacteria, Bacteroidetes, Planctomycocetes, Proteobacteria, and Verrucomicrobia, comprising 90.98% of the total relative abundance (Figures 5A,B). The remaining identified phyla (22) showed a relative abundance lower than 3% and were considered rare phyla. Confronting the relative abundance between plant groups, four phyla (Fibrobacteres, Gemmatimodetes, Planctomycetes, and Verrumicrobia) showed significantly higher relative abundance in the rhizoplane of the hyperaccumulator compared to the non-hyperaccumulators (Figure 5A). Additionally, two phyla (BRC1 and OP11) were absent on the non-hyperaccumulator rhizoplane but were present on the hyperaccumulator. Hereafter, we refer to these as “exclusive taxa” of *N. brachypetala* (Figure 5A). Three more phyla (Acidobacteria, Elusimicrobia, and WS3) were more abundant in the rhizosphere of the hyperaccumulator. In contrast, the phyla Fibrobacteres and Proteobacteria exhibited increased abundance in the non-hyperaccumulator rhizosphere and rhizoplane, respectively. The phylum Fibrobacteres was in a higher abundance in the rhizosphere of the non-hyperaccumulator and, at the same time, had a larger presence in the rhizoplane of the hyperaccumulator. To sum up, six rare and three dominant phyla differed between *N. brachypetala* and the non-hyperaccumulators in both root compartments.

Regarding the fungal domain, the dominant phyla were Ascomycota (53%) and Basidiomycota (36%) (Figures 5C,D). The remaining identified phyla (10) had a combined relative abundance of less than 3% (Figure 5C). The relative abundance of each sample is provided in Supplementary Figure 3. The hyperaccumulator harbored two exclusive fungal phyla (Mucoromycota and Olpidiomycota) in the rhizoplane and one (Monoblepharomycota) in the rhizosphere (Figure 5C). The major phyla were in different composition according to the plant host. Ascomycota were more abundant in the rhizosphere of the hyperaccumulator, whereas Basidiomycota had a larger presence in the rhizosphere of the non-hyperaccumulators. Conversely, in the non-hyperaccumulators, three phyla were more abundant in their rhizoplane (Chytridiomycota, Kickxellomycota, and Monoblepharomycota) and one (Basidiomycota) in the rhizosphere. Additionally, six minor phyla were composed differently between the plant species.

Generally, the taxa abundance by prevalence revealed that the most abundant bacteria were also the most prevalent. In contrast, the most abundant species in the fungal domain exhibited a lower prevalence (Supplementary Figure 2).

### The Bacterial Community of *N. brachypetala* Is Globally More Diverse With Higher Relative Abundance in the Rhizoplane Compartment

**FIGURE 6:**
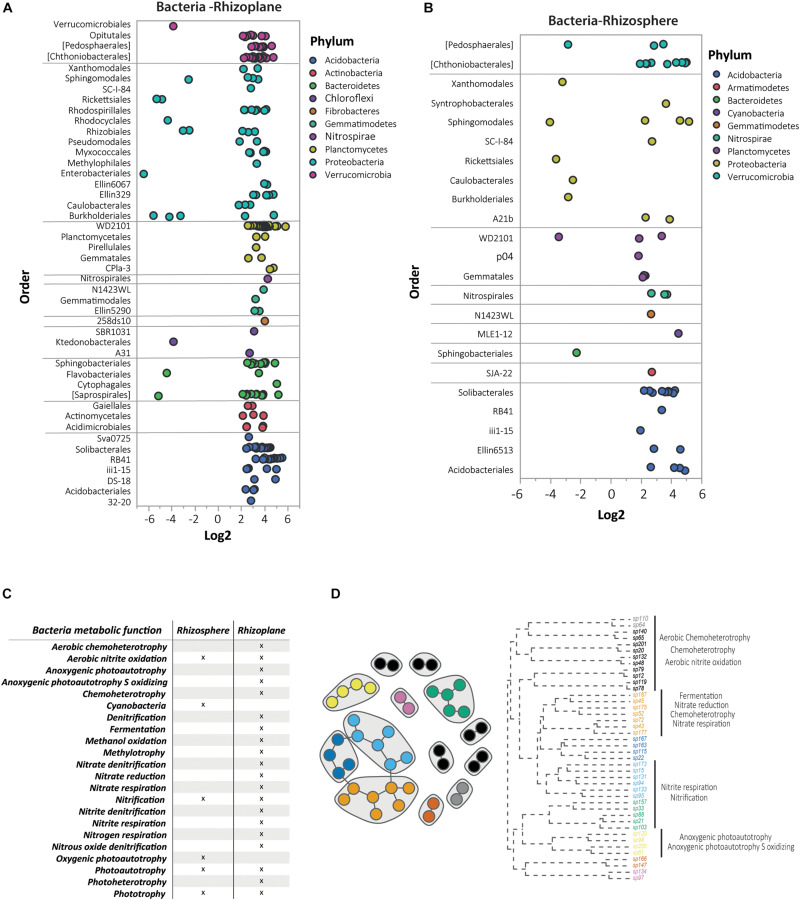
Differential abundance analysis (DAA) and metabolic functions of bacterial OTUs in *N. brachypetala* and non-hyperaccumulator species. Bubble plots of the differential abundant bacterial OTUs on the rhizoplane **(A)** and rhizosphere **(B).** The *x* axis indicates the log2 ratio (hyperaccumulators vs. non-hyperaccumulators). The *y* axis indicates bacteria at the taxonomical level of order. Bubble colors indicate the associated phylum. **(C)** The co-occurrence network of the DAA bacterial OTUs in the rhizoplane of *N. brachypetala* and a dendrogram with the annotated functions. **(D)** Table of metabolic functions of the DAA bacteria in *N. brachypetala* in each root compartment.

To gain further insight into the taxa distribution, the bacterial OTUs were subjected to a DAA comparing *N. brachypetala* with the non-hyperaccumulator species (Figure 6). The DAA results were presented at the taxonomic level of order, the lowest level that was visually informative. The bacterial analyses of the rhizoplane showed an important accumulation of dots with positive log2 values, indicating a higher abundance of bacteria (by order) in *N. brachypetala* (Figure 6A). The highest number of differentially abundant bacteria belonged to five dominant phyla (Acidobacteria, Bacteroidetes, Planctomycetes, Proteobacteria, and Verrucomicrobia). Four rare phyla (Chloroflexi, Fibrobacteres, Gemmatimonadetes, and Nitrospirae) were also abundant in the hyperaccumulator rhizoplane. At the taxonomic level of family, *N. brachypetala* presented 108 families in the rhizoplane and 112 in the rhizosphere with a higher relative abundance than in the non-hyperaccumulator species (Supplementary Dataset 3). The number of more abundant families associated with the non-hyperaccumulator species was in an order of magnitude lower, of 20 and 15 in the rhizoplane and rhizosphere, respectively. The most prevalent taxa appearing repetitively were *Candidatus Solibacter* and the genera *Flavobacterium*, *Koribacter*, *Opitutus*, *Planctomyces*, *Pseudomonas*, and *Streptomyces* (Supplementary Dataset 3).

The DA OTUs from *N. brachypetala* were explored to identify their metabolic functions (Figures 6C,D). Functional analysis of the hyperaccumulator bacteria identified 22 metabolic functions. The most representative functions were those related to the nitrogen cycle (Figure 6C). The rhizoplane with 20 of 22 obtained functions was the most diverse functional root compartment. Rhizoplane bacteria were involved in functions related to the nitrogen cycle, whereas the rhizosphere bacteria were exclusively linked to cyanobacteria and oxygenic photautotrophy (Figure 6C). The interaction network in the DA bacteria of the rhizoplane and the associated functions are provided in Figure 6D. The observed pattern revealed that interconnected bacteria were involved in several metabolic functions. Aerobic nitrite oxidation was assigned to isolated bacteria (black and gray dots), with the nitrification and nitrite respiration assigned to non-connected bacteria (blue and green dots). Therefore, the functions were not diversified within the connected groups.

### The Fungal Community of *N. brachypetala* Presents a Reduced Differential Abundance Compared to the Non-hyperaccumulator Species

**FIGURE 7:**
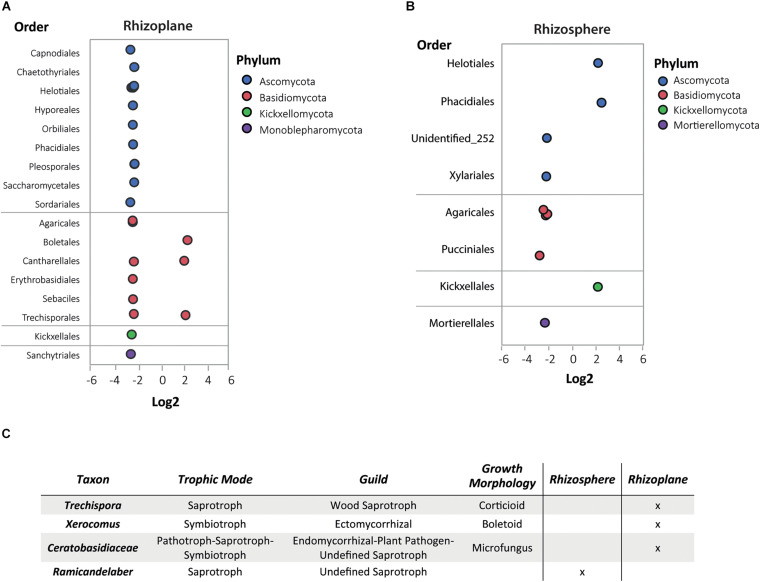
Differential abundance analysis (DAA) and guilds of fungal OTUs in *N. brachypetala* and non-hyperaccumulator species. Bubble plots of the differential abundant fungal OTUs in the rhizoplane **(A)** and rhizosphere **(B).** The *x* axis indicates the log2 ratios (hyperaccumulators vs. non-hyperaccumulators). The *y* axis indicates fungi at the taxonomical level of order. Bubble colors indicate the associated phylum. **(C)** Table of trophic mode and guilds of DAA fungi in *N. brachypetala* in each root compartment.

The DAA of the fungi revealed more abundant fungi (by order) in both root compartments of the non-hyperaccumulator species (Figures 7A,B). Some of the limited fungal species in the rhizoplane of *N. brachypetala* were *Candida railenensis*, *Clarireedia bennettii*, *Oidiodendron chlamydosporicum*, *Trechispora byssinella*, and *Acremonium rutilum* (Supplementary Dataset 3).

Conversely, we also detected seven fungi OTUs exclusive to the *N. brachypetala* plants (Supplementary Dataset 3). To our knowledge, there are no tools available to classify fungal communities into metabolic functional groups, but FUNGuild (Nguyen et al., 2016) predicts function at the guild level. Figure 7C indicates that the four fungal OTUs exhibit different trophic strategies. Among them, the genus *Xerocomus* deserves highlighting because it is a symbiont described as an ectomycorrhiza.

## Discussion

Microbiota associated with plant roots are considered important mediators of plant–soil interactions. In the case of metal hyperaccumulator plants, rhizosphere bacteria have been associated with positive impacts on plant growth and increased metal accumulation (Visioli et al., 2015a). Therefore, the root microbiome of hyperaccumulators has attracted increased attention for potential biotechnological applications in phytoremediation. Despite the interest, there is little information on the root–microbiota communities and their associated functions in most hyperaccumulators (Thijs et al., 2017). Additionally, attention has been focused on hyperaccumulators from metalliferous soils, and those inhabiting non-polluted soils remain practically unexplored. Our study aimed to identify the bacterial and fungal taxa and their functional attributes associated with *N. brachypetala* plants from non-metalliferous soils. Microbia identification of field samples was performed using high-throughput sequencing in order to identify novel taxa that could be involved in metal hyperaccumulation in a cultivation-independent manner.

According to our results, the hyperaccumulator, *N. brachypetala*, exhibited an increased richness in the bacterial community compared to the companion non-hyperaccumulator Brassicaceae species. This hyperaccumulator also presented the most complex and highly interconnected interaction network. In our study, the rhizoplane of *N. brachypetala* harbored a higher relative abundance of bacterial OTUs, with distinct rare taxa. Low-abundance taxa have been recognized to provide critical functions in ecosystems and represent a vast functional gene pool (Jousset et al., 2017; Compant et al., 2019; Xiong et al., 2020). Therefore, the identification of satellite groups associated with the root niche of a hyperaccumulator plant growing on natural and undisturbed soil is highly interesting. For example, Nitrospiraceae and Gemmatimodaceae (which are more abundant in the rhizoplane of *N. brachypetala*) have been previously associated with the rhizosphere of the Cd/Zn hyperaccumulator *A. halleri* (Muehe et al., 2015). The metal(loid)-tolerant phylum Chlamydiae (Schneider et al., 2017) also increased in the rhizoplane of our hyperaccumulator. Additionally, the rhizosphere of *N. brachypetala* includes Thermogemmatisporaceae, which was formerly detected in high concentrations in the reclaimed soil of an ancient mine (Pershina et al., 2020), and Moraxellaceae, which includes heavy metal resistant species (Chen et al., 2015).

Ultimately, there is a substantial variation in the consortia of microbes in the taxonomically related plants that cohabit the undisturbed soils, but differ in the hyperaccumulator phenotype. Considering that the cited microbial taxa are much less abundant in the soil fraction associated with non-hyperaccumulator plants, our results suggest a selection of metal-tolerant bacteria exist in the root fractions of *N. brachypetala*. The metal tolerance of microbia colonizing the rhizosphere of hyperaccumulators in common soils was not the focus of this research. Therefore, it is an interesting topic for future studies on microbial cultivation.

Our results from the DAA identified *Candidatus Solibacter* (Acidobacteria) as one of the most prevalent bacteria in the rhizoplane of *N. brachypetala* growing on non-metalliferous soil. *C. Solibacter* has been identified in the core of the bacterial community in soils polluted with heavy metals (Golebiewski et al., 2014; Wang et al., 2018). *Kaistobacter* (which is associated with hyperaccumulators) has been similarly described in contaminated soils (Wu et al., 2019). Another abundant genus was *Streptomyces*, which has been previously affiliated with the plant *Sedum alfredii* in Cd-polluted soil (Hou et al., 2018). We also identified *Flavobacterium* with a high DAA ratio, which has been associated with metal accumulation in different hyperaccumulators (Idris et al., 2004; Luo et al., 2011, 2017; Zhang et al., 2012). Any of the differentially abundant bacteria of *N. brachypetala* may be assigned as metal solubilizers because the applied software (FAPROTAX) did not include this functional category.

A distinctive functional trait of the bacterial communities in *N. brachypetala* is their role in the nitrogen cycle. This function is not a direct mechanism of metal acquisition or tolerance. However, increased nitrogen availability can be advantageous for plants growing in metal-polluted soils. Nitrate assimilation is reduced under moderate metal exposure, confirming that metal toxicity alters nitrogen uptake by plants (Wang et al., 2008). Interestingly, co-cropping of legumes with hyperaccumulators has been reported to improve soil quality and fertility and increase metal yield (Saad et al., 2016, 2021). Our results provide a potential specialized rhizosphere bacterial community that facilitates N recycling. The plants in our study inhabited non-polluted sites; therefore, bacterial selection should occur regardless of the soil metal content. To confirm this hypothesis, it will be necessary to study the microbial community of *N. brachypetala* under metal exposure.

Our results identified bacteria contrasts with the DAA ratios for mycobiota between hyperaccumulator and non-hyperaccumulator species. Fungal species were generally much more enriched in the non-hyperaccumulator species than in the hyperaccumulator. However, it cannot be concluded that *N. brachypetala* was globally deficient in fungi, as the relative abundance for all plant species was high. Hyperaccumulator plants harbored seven exclusive fungal taxa. Nonetheless, certain specific fungal biota were absent in the hyperaccumulator. If we assume that the distribution of the root-associated microorganisms is not a hazardous process, we can speculate a potential positive selection in the rhizoplane of *N. brachypetala* toward certain bacteria with a detrimental effect on specific fungal groups. Among the exclusive fungi in the hyperaccumulator, *Xerocomus* could positively affect the functioning of *N. brachypetala* as it is a symbiotroph annotated as ectomycorrhizal species. Ectomycorrhizal fungi can help the hyperaccumulator phenotype through chemical immobilization in the cell wall or complexation with organic exudations (Luo et al., 2014). The Brassicaceae family has a high percentage of non-mycorrhizal species (Wang and Qiu, 2006), but with exceptions such as *N. praecox* (a Zn, Cd, and Pb hyperaccumulator), which, under field conditions, is colonized by mycorrhiza during the developmental stages that are coincident with low glucosinolate levels (Vogel-Mikus et al., 2005; Pongrac et al., 2008). Our DA analysis revealed that *Xerocomus subtomentosus* was 100 times more abundant in the rhizoplane of *N. brachypetala*. However, the confirmation of functional root colonization requires further analysis. To the best of our knowledge, any of these increased fungal taxa have been previously related to metal-polluted soils or hyperaccumulator species. Only *A. rutilum* and *O. chlamydosporicum* have been described as tolerant to abiotic stresses such as salinity and acidic pH, respectively (Fritze and Baath, 1993; Georgieva et al., 2012). These taxa deserve further investigation for their potential contributions to the hyperaccumulation phenotype.

Our results demonstrated the root compartment (rhizosphere vs. rhizoplane) and plant host (*N. brachypetala* vs. non-hyperaccumulators) were the major factors influencing the microbiome diversity, structure, and composition. Previous studies have revealed that microbial communities are strongly influenced by plant roots increasing toward the root (Bulgarelli et al., 2012; Lundberg et al., 2012; Martinez-Diz et al., 2019; Thiergart et al., 2020). In contrast, the host plant is suggested to be a weaker predictor of root–microbiota composition (Schlaeppi et al., 2014; Glynou et al., 2018; Macia-Vicente et al., 2020). However, host influence has mainly been evaluated among genotypes of a specific plant species. Consequently, studies addressing the role of host identity in harboring different microbial species are required. Recent field research indicates that host identity in comparing different plant species is a strong variable in the determination of microbial assembly (Burns et al., 2015; Borymski et al., 2018; Li et al., 2018; Wang et al., 2020).

Previous studies have indicated that the composition of root-inhabiting microbiome communities is highly influenced by soil type (Bulgarelli et al., 2012; Lundberg et al., 2012; Schlaeppi et al., 2014) and geographical location (Glynou et al., 2018; Lin et al., 2020). Nonetheless, both factors affect the root-associated microbes in a domain-dependent manner, with a stronger effect of soil properties on bacteria and the geographic location of fungi (Thiergart et al., 2020). In our research, the study location was not a significant factor in the microbial community variations. Further large-scale research with more populations from distant locations should be conducted to confirm this result. The Pyrenees are the southern limit of *N. brachypetala*, where the species is rare and in regression (Martos et al., 2016); future studies should consider researching Northern populations.

Root–microbial communities play a pivotal role in plant performance by improving mineral nutrition (Jacoby et al., 2017). Bacteria and fungi have been reported to contribute to the use of major nutrients such as N, P, and S (Hong et al., 2015; Almario et al., 2017; Perez-Izquierdo et al., 2019; Zhang et al., 2019). Microbes have also been involved in the cycling of some micronutrients, mainly Fe, Zn, and Mn (Alegria Terrazas et al., 2016). In our study, some soil elements (Ca and K) made a minor but significant contribution to the microbial assembly. To the best of our knowledge, the role of these elements in the composition and structure of root microbes has yet to be investigated.

In conclusion, our results comparing non-hyperaccumulator and hyperaccumulator species indicate that the root compartment and the plant host are key factors influencing the diversity, structure, and composition of root-associated bacteria. The fungal community was mainly determined by the root niche, but not by the host. Conversely, location was an irrelevant factor in any microbial group variation. We observed a global reduction in microbial α diversity in the rhizoplane. *N. brachypetala* was a notable exception, presenting a prominent diversity of bacteria forming a complex interaction network in the closest-root compartment. *N. brachypetala* harbored a large group of differentially abundant bacteria, whereas fungi were more dominantly associated with the non-hyperaccumulator species. Even though both the non-hyperaccumulator and the hyperaccumulator inhabited non-metalliferous soils, we detected enriched levels in the *N. brachypetala* root compartments of bacteria previously described as metal-tolerant or formerly associated with hyperaccumulators in metal-rich soils. In contrast, the hyperaccumulator was impoverished in certain fungi. Interestingly, we found that most of the differentially abundant bacteria in the hyperaccumulator were involved in the nitrogen cycle. Our results suggest a potential selection of beneficial taxa in the soil fractions influenced by the roots of *N. brachypetala* that can favor N availability to the plants, indirectly contributing to their hyperaccumulator phenotype.

## Data Availability Statement

All sequence data are freely available in the European Nucleotide Archive database (Project PRJEB41937) and will be available upon publication.

## Author Contributions

CP conceived the study and revised the manuscript. SM designed the study and wrote the manuscript. SB, ML, and CC performed the field sampling work. LP-M and SB analyzed the data. All authors approved the final manuscript.

## Conflict of Interest

The authors declare that the research was conducted in the absence of any commercial or financial relationships that could be construed as a potential conflict of interest.

## Supplementary Material

Supplementary Figure 1 Rarefaction curves.

Supplementary Figure 2 Prevalence for bacteria and fungi.

Supplementary Figure 3 Relative abundance per sample.

Supplementary Dataset 1 Sample information and sequencing quality. Stats for RP1 and RP2.

Supplementary Dataset 2 Soil and plant leaf ions composition and statistics.

Supplementary Dataset 3 List of bacterial and fungal taxa according to the differential abundance analysis (DAA) within each root compartment.
