# Supplementary figures and images for: Identifying the Specific Root Microbiome of the Hyperaccumulator Noccaea brachypetala Growing in Non-metalliferous Soils
Source: Front Microbiol. 2021 May 14;12:639997. doi: 10.3389/fmicb.2021.639997 (PMC8160108; doi:10.3389/fmicb.2021.639997)

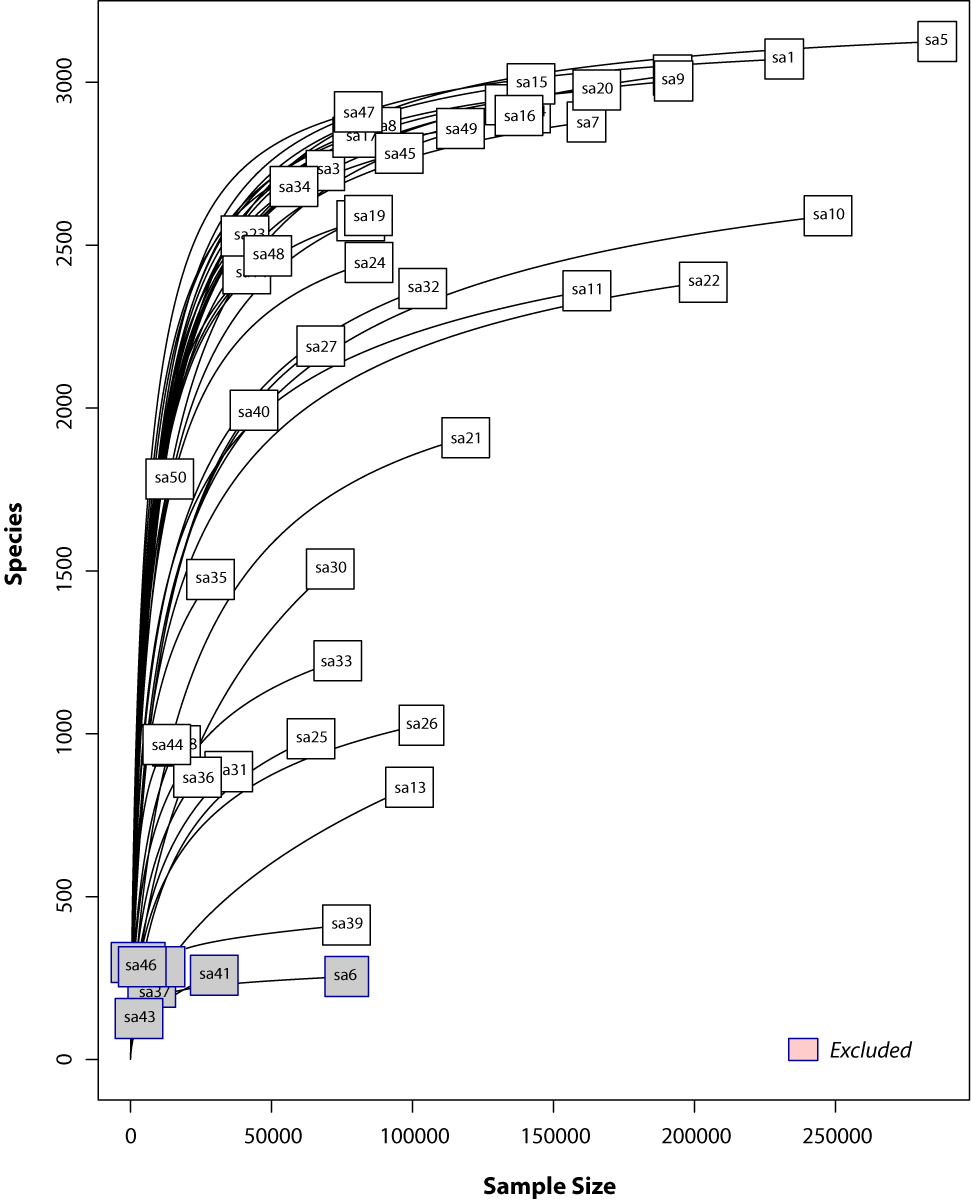

Supplement: Supplementary Figure 1 — Rarefaction curves. [file Image_1.TIF]

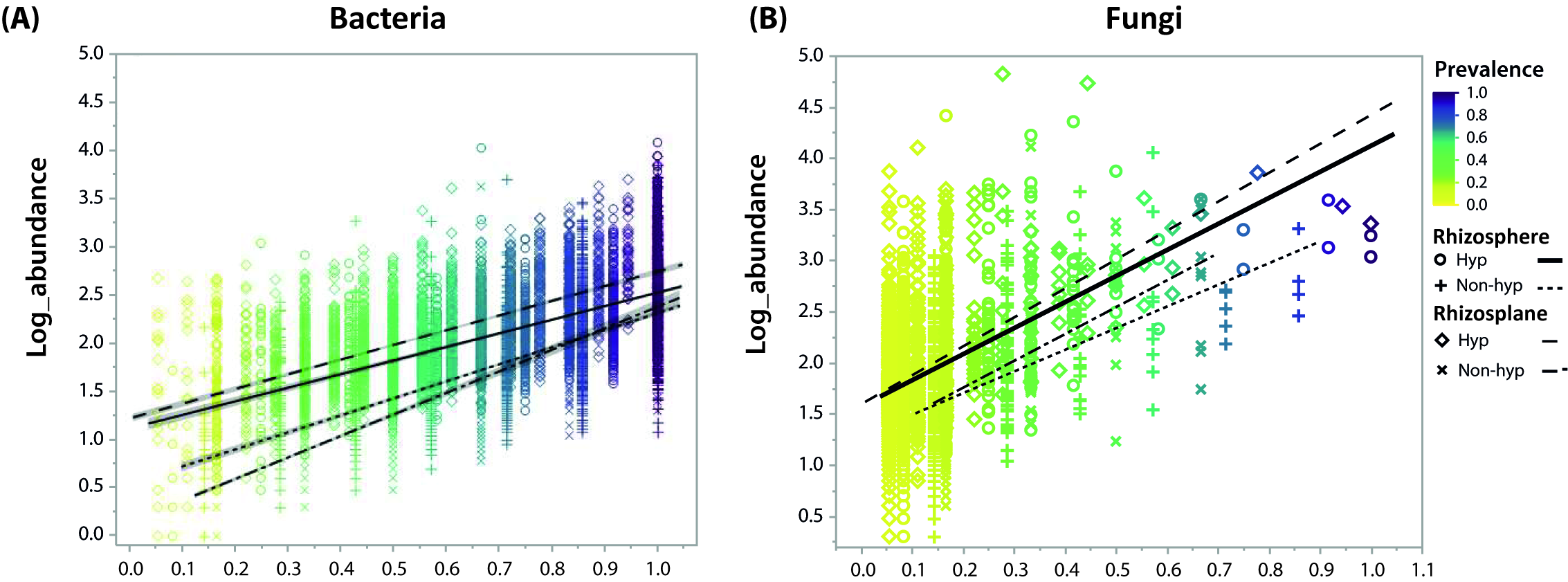

Supplement: Supplementary Figure 2 — Prevalence for bacteria and fungi. [file Image_2.TIF]

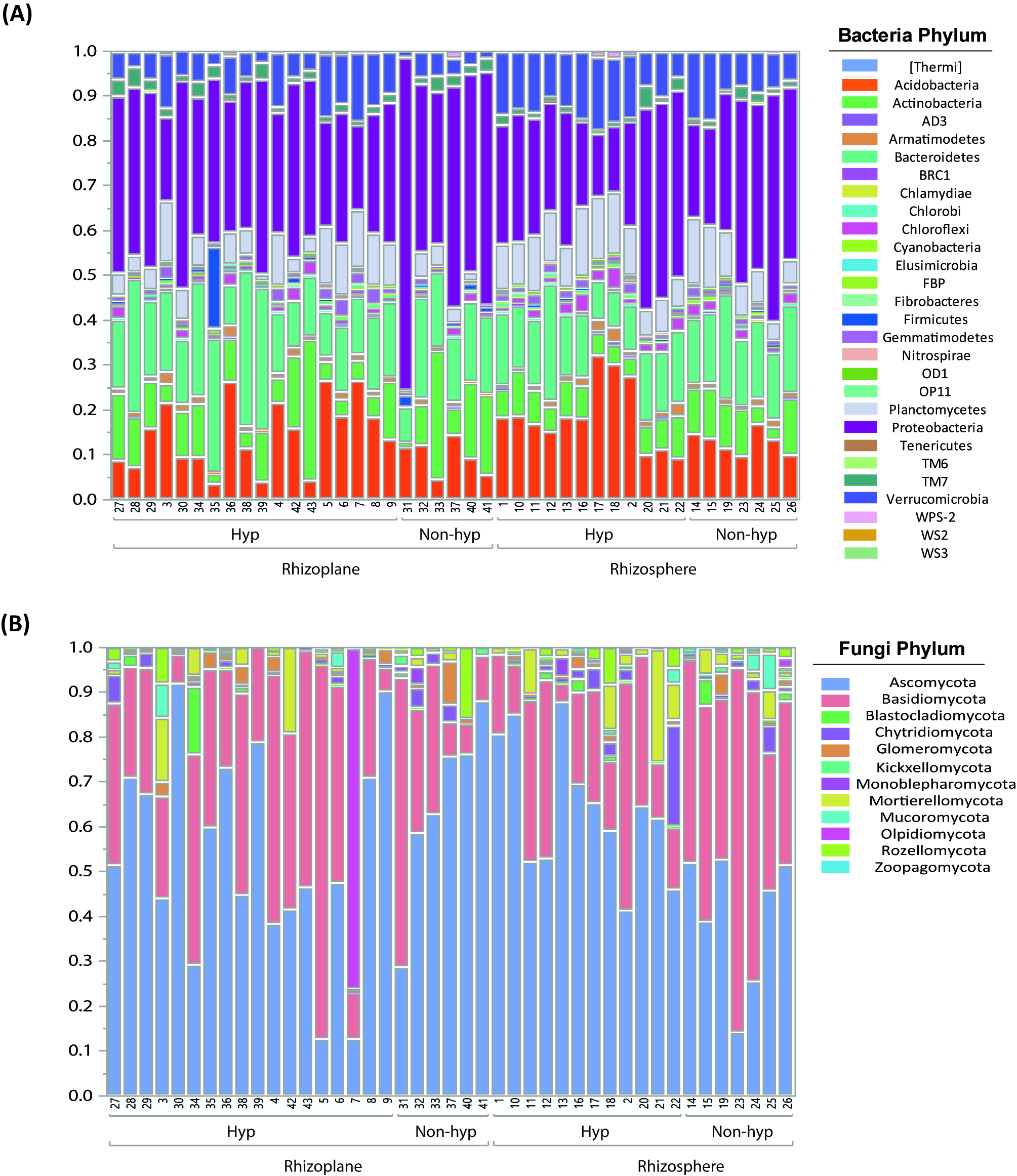

Supplement: Supplementary Figure 3 — Relative abundance per sample. [file Image_3.TIF]
